# Splenic cystic lymphangioma mimicking hydatid cyst in an adult: a rare diagnostic challenge

**DOI:** 10.1093/jscr/rjag174

**Published:** 2026-03-20

**Authors:** Hussain Alessa, Tahar Yacoubi, Jawaher Alraihan, Ibrahim Alhusain, Fahad Elmokyed, Falah Alotaibi, Omar Almakhayitah, Omar Alkhaldi, Abdullah Al Ghamdi

**Affiliations:** Department of General Surgery, King Abdulaziz Hospital, King Saud Road, Almubarraz District, Alahsa 39182, Saudi Arabia; Department of Pathology and Lab Medicine, King Abdulaziz Hospital, King Saud Road, Almubarraz District, Alahsa 39182, Saudi Arabia; Department of General Surgery, King Abdulaziz Hospital, King Saud Road, Almubarraz District, Alahsa 39182, Saudi Arabia; Department of General Surgery, King Abdulaziz Hospital, King Saud Road, Almubarraz District, Alahsa 39182, Saudi Arabia; Department of General Surgery, King Abdulaziz Hospital, King Saud Road, Almubarraz District, Alahsa 39182, Saudi Arabia; Department of General Surgery, King Abdulaziz Hospital, King Saud Road, Almubarraz District, Alahsa 39182, Saudi Arabia; Department of General Surgery, King Abdulaziz Hospital, King Saud Road, Almubarraz District, Alahsa 39182, Saudi Arabia; Department of General Surgery, King Abdulaziz Hospital, King Saud Road, Almubarraz District, Alahsa 39182, Saudi Arabia; Department of General Surgery, King Abdulaziz Hospital, King Saud Road, Almubarraz District, Alahsa 39182, Saudi Arabia

**Keywords:** spleen, lymphangioma, cysts, open splenectomy, rare tumor

## Abstract

Splenic cystic lymphangioma is a rare, benign disease affecting the splenic lymphatic system; it is uncommon in adults and primarily seen in children. Symptoms are usually nonspecific. The diagnosis is confirmed by histopathological analysis. We report a 58-year-old female patient with left upper abdominal pain and a history of abdominal trauma with mild tenderness over the left upper quadrant. Abdominal ultrasound revealed a well-defined multilocular cystic lesion in the spleen, measuring ~7.9 × 6.9 cm. Then, abdominal and pelvic computed tomography revealed a large, non-enhancing hypodense cystic lesion in the spleen with internal septations, calcifications, and multiple smaller daughter cysts arranged peripherally; hydatid cyst was the primary differential diagnosis. After that, she underwent an open splenectomy under general anesthesia. Final pathology results confirming a benign splenic cystic lymphangioma. Splenic cystic lymphangiomas are rare, non-malignant tumors that are infrequently diagnosed in adults. Surgical removal via splenectomy is the treatment of choice.

## Introduction

Splenic cystic lymphangioma is a rare benign disease that affects the splenic lymphatic system, resulting in dilation of the lymphatic vessels; it is mostly seen in children [1, 2]. Adult occurrences of splenic lymphangioma are relatively uncommon, with 80% to 90% of cases occurring in children [3]. Patients can present with small lesions (<5 cm) that are often detected through imaging studies or large, complex lesions that compress nearby organs [4]. Abdominal pain, fever, fatigue, weight loss, and hematuria are among the clinical manifestations of lymphangiomas. But often, it is asymptomatic [5, 6].

It is usually incidentally detected through abdominal computed tomography or ultrasonography [1]. On computed tomography (CT) scans, splenic lymphangiomas typically appear as thin-walled cystic masses with minimal to no enhancement of the thin septa [7]. Histopathological analyses typically yield the final diagnosis [8]. To avoid significant consequences, the standard of care is total surgical removal of the tumor [9].

In this article, we report a challenging case of a 58-year-old female patient with left upper quadrant pain, for which the initial diagnosis raised the possibility of a hydatid cyst, which then turned out to be splenic cystic lymphangioma, which was confirmed by histopathology after splenectomy. Our aim is to report an additional case of splenic cystic lymphangioma.

## Case presentation

A 58-year-old female, known to have osteoarthritis of the knees, dyslipidemia, and vertigo, with no past surgical history and no known allergies. She was admitted through the emergency department under general surgery due to the patient having a complaint of intermittent left upper quadrant abdominal pain, dull in nature, not radiated, and associated with nausea without vomiting. As well, she gave a history of abdominal trauma previously. Other systematic reviews were unremarkable. Upon examination the patient had appeared conscious, alert, and oriented to time, place, and person; she was vitally stable and afebrile. Her abdomen was soft and lax with mild tenderness over the left upper quadrant on deep palpation. No organomegaly was detected, and no palpable mass was appreciated.

**Figure 1:**
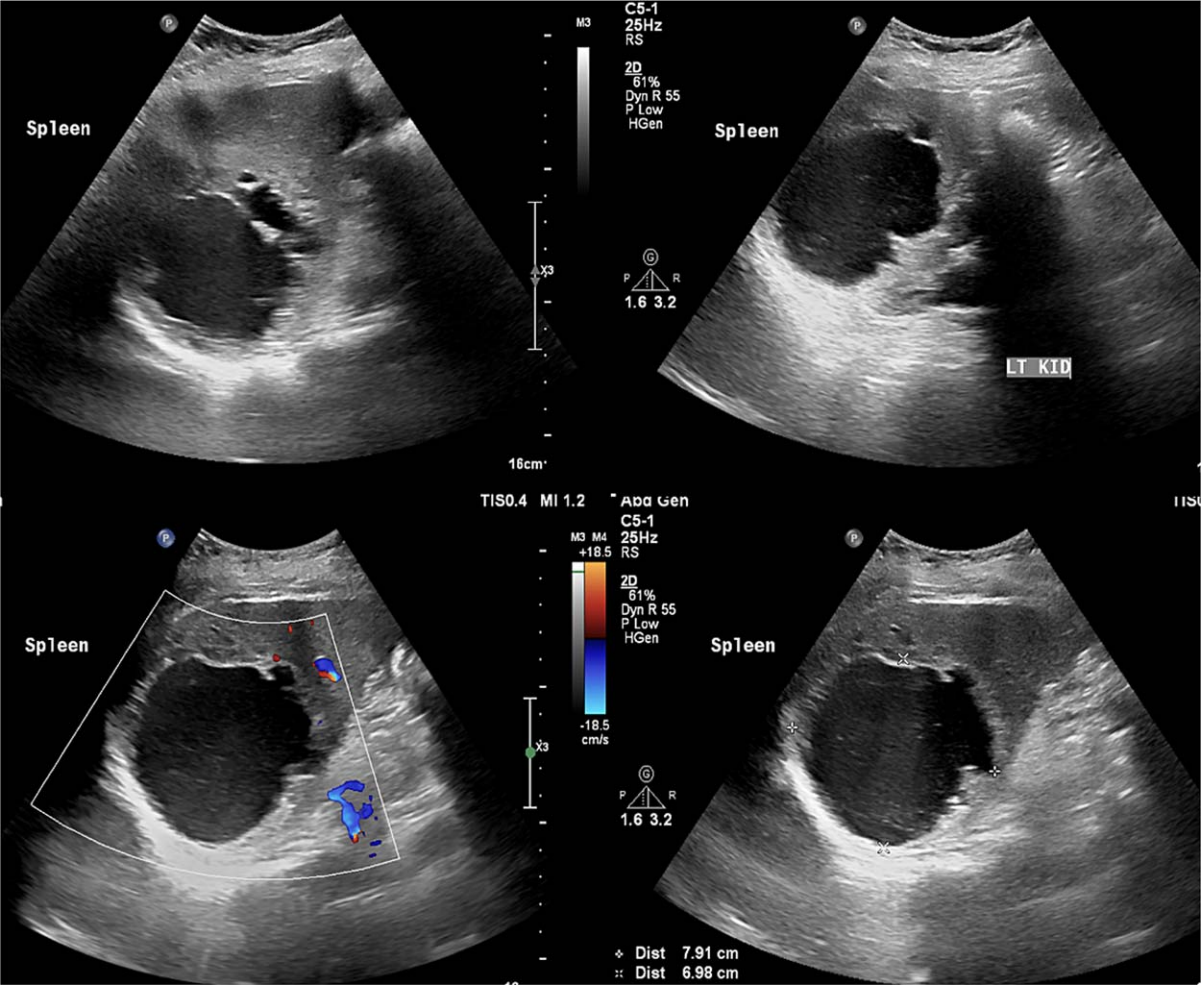
Abdomen ultrasound revealed well-defined multilocular cystic lesion with internal echoes in the spleen, measuring ~7.9 × 6.9 cm.

**Figure 2:**
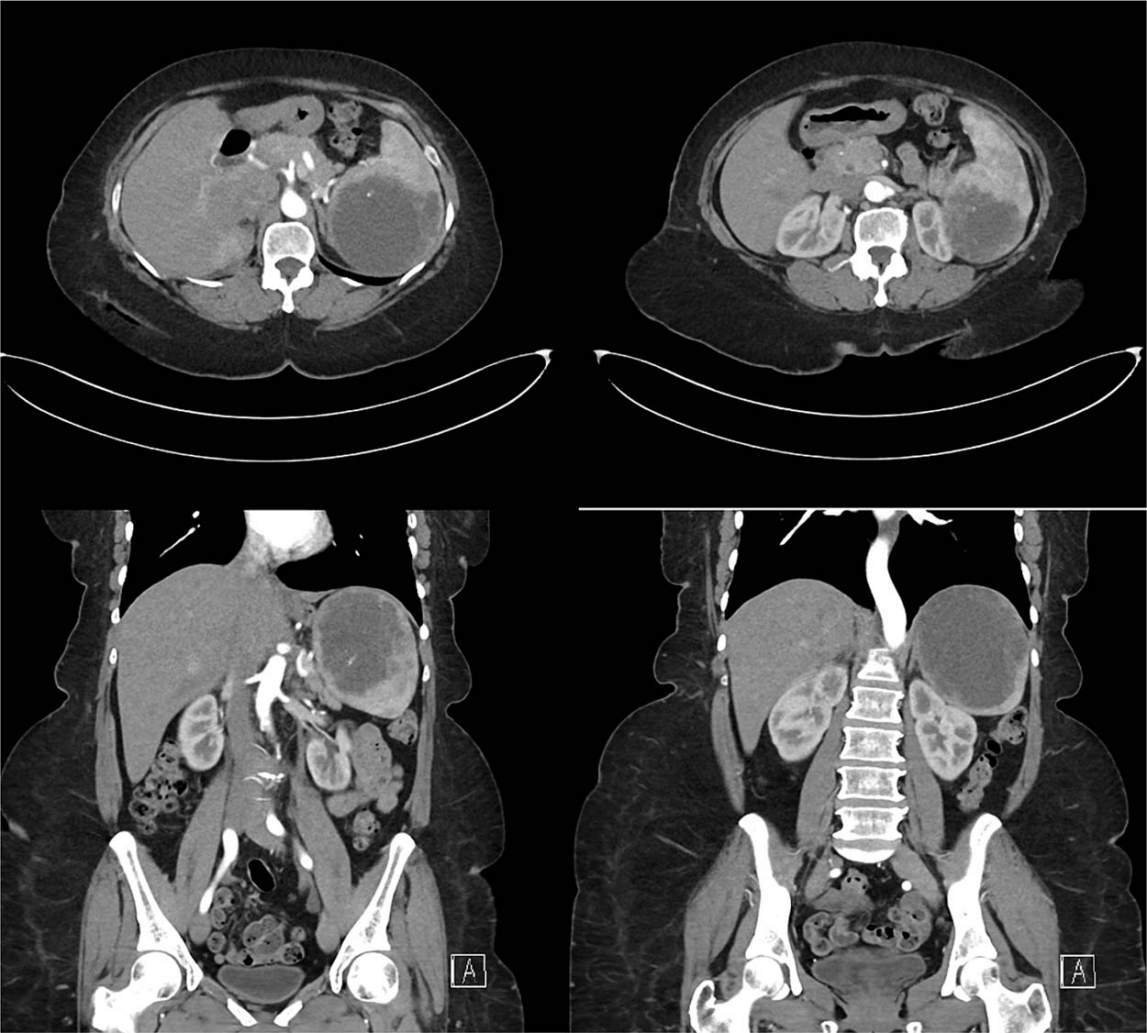
Abdominal and pelvic CT scan revealed a large, non-enhancing hypodense cystic lesion in the spleen with internal septations, calcifications, and multiple smaller daughter cysts arranged peripherally. Prime differential was type II hydatid cyst.

Laboratory results were within normal limits (hemoglobin 120 g/l, WBC 4.72 × 10^9^/l). Abdominal ultrasound showed a well-defined multilocular cystic lesion with internal echoes in the spleen, measuring 7.9 × 6.9 cm, with possible hematoma (Fig. 1). No significant free fluid was detected. A CT scan revealed a large, non-enhancing hypodense splenic cyst with internal septations, calcifications, and multiple peripheral daughter cysts (Fig. 2). The splenic hilum and capsule were intact, with no perisplenic fat stranding. The main differential diagnosis was a type II hydatid cyst. No solid organ injury or significant free fluid was observed.

The infectious diseases team was consulted regarding the patient’s state, and they concluded that medical therapy alone would not be sufficient for this patient and recommended exploring surgical options, with Albendazole to be considered once a definitive treatment plan was established. The patient was subsequently referred to the outpatient clinic for the necessary medications and vaccines (pneumococcal vaccines, meningococcal vaccines, and *Haemophilus influenzae* type b) before surgery for a suspected splenic hydatid cyst. Further investigations were performed to rule out hydatid disease. Echinococcus antibody serology was negative, and viral hepatitis screening showed HBsAg, anti-HBc, and anti-HBs were all non-reactive.

The patient underwent an open splenectomy through a left subcostal incision, which revealed an enlarged spleen and that the splenic artery was initially ligated. Due to radiologic suspicion of a type II hydatid cyst, scolicidal-soaked gauze and careful handling were used to avoid rupture.

**Figure 3:**
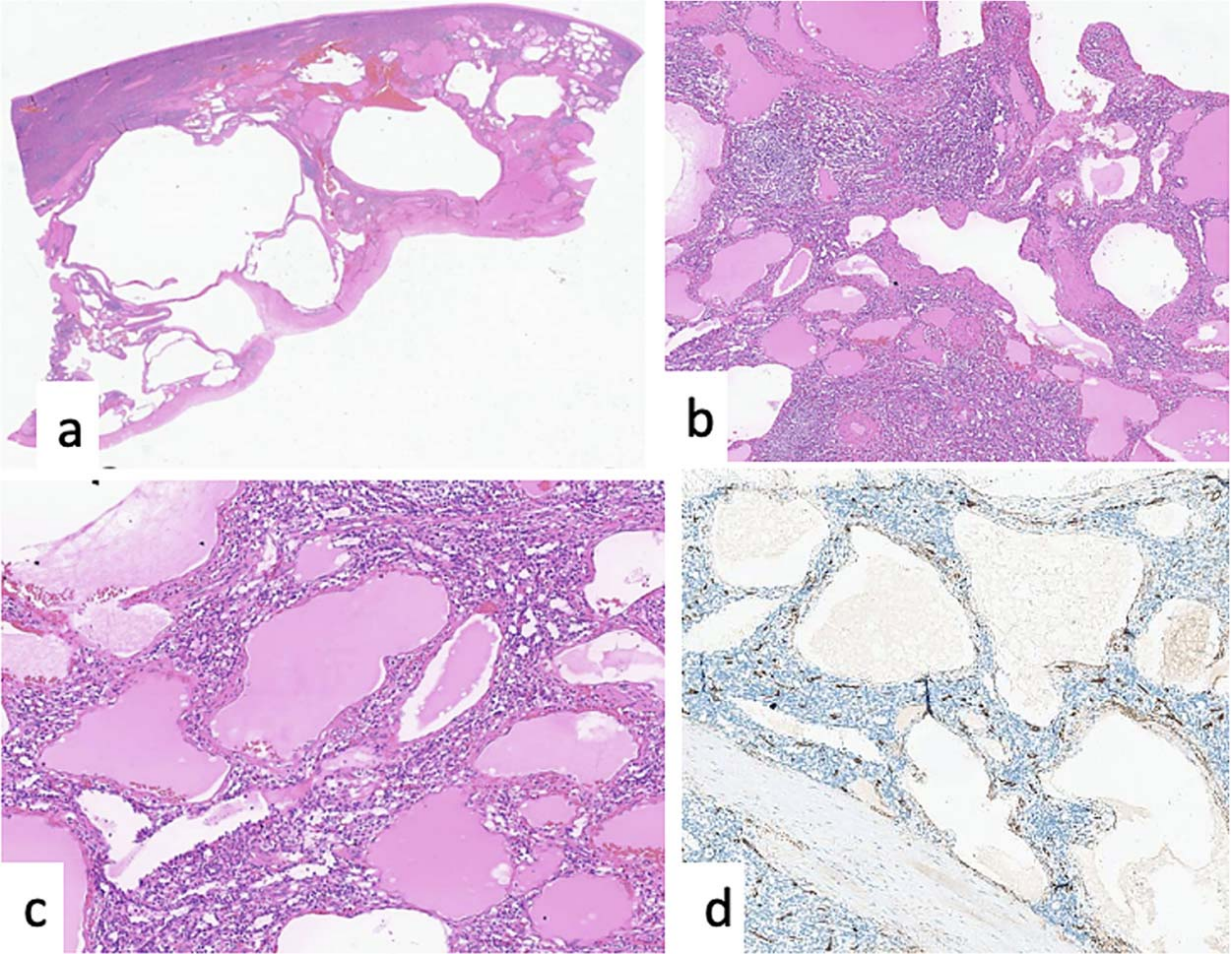
Cystic lymphangioma of the spleen. (a) HE × 20: ill-defined cystic lesion. (b, c) HE × 40, HE × 100: vessels of different caliber and shape filled by eosinophilic materiel. (d) Positivity of CD34 in the endothelial cells lining the lymphatic vessels.

Postoperatively, the patient tolerated oral intake; the catheter and nasogastric tube were removed on Day 1, and the drain on Day 5. She was discharged on Day 8 in good condition. Pathology confirmed a benign splenic cyst (cystic lymphangioma) (Fig. 3). At follow-up, she was asymptomatic and ambulating independently, and albendazole was discontinued.

## Discussion

Splenic cystic lymphangioma is an extremely rare benign lymphatic disease, primarily seen in children, with adult cases being rare [10]. It represents 0.007% of cancers overall; it could also present as a component of systemic lymphangiomatosis syndromes that could involve splenic lymphangiomas [11]. The rarity of this condition in adults makes its diagnosis a challenge, and it is frequently diagnosed in conjunction with other solid and cystic splenic lesions, such as hemangiomas, chronic infections, lymphomas, and metastases, with solid and cystic sections that can occasionally be seen in hemangiomas [12]. Only nine occurrences of isolated splenic lymphangiomatosis were documented between 1990 and 2010 [13].

Most splenic lymphangiomas are asymptomatic and are incidentally detected during imaging for unrelated conditions [14]. Patients are either asymptomatic or exhibit symptoms such as nausea, loss of appetite, abdominal distension, or pain in the left upper quadrant. Sometimes splenomegaly is accompanied by the compression of nearby organs, in addition to presenting complications such as portal hypertension, hypersplenism, hemorrhage, or consumptive coagulopathy [15]. This relates to our patient, who only complained of intermittent left upper abdominal pain with mild tenderness on deep palpation.

CT is a key diagnostic tool for splenic lymphangioma because it provides detailed features, including wall thickness, internal septations, and tiny mural nodules. Ultrasound can reveal calcifications and multiple hypoechoic cysts with hyperechoic septa [16]. On MRI, they typically appear as multilocular cystic lesions with thin septations, hypo-intense on T1-weighted scans and hyperintense on T2-weighted images, corresponding to dilated lymphatic channels [17].

Depending on the congenital dilated lymphatic channels, lymphangiomas can be histologically classified into three subtypes: capillary (super-microcystic), cavernous (microcystic), or cystic (macrocystic) [12]. In our case, pathology confirmed the presence of a benign cystic lymphangioma after surgical removal.

Total splenectomy remains the gold standard for treating these lesions, preventing complications such as bleeding, hypersplenism, rupture, or malignancy [18]. The choice between open and laparoscopic approaches depends on splenic size, patient factors, and surgeon experience. Laparoscopy is preferred for normal to moderately enlarged spleens, while open surgery is favored for significantly enlarged spleens due to technical challenges and higher intraoperative risk [19]. In our patient, the open approach was chosen because of the lesion’s size.

## Conclusion

Splenic cystic lymphangioma is uncommon in adults; it is thought to be considered as a differential diagnosis for splenic cystic lesions. CT scans are the key diagnostic modality to ensure accurate characterization of splenic cystic lesions, while ultrasounds may be helpful as an initial assessment tool. But for accurate diagnosis, the surgically removed specimen must undergo pathological analysis.
